# Surface Modification of Polymer Substrates for Biomedical Applications

**DOI:** 10.3390/ma10101115

**Published:** 2017-09-21

**Authors:** Oldřich Neděla, Petr Slepička, Václav Švorčík

**Affiliations:** Department of Solid State Engineering, University of Chemistry and Technology, 166 28 Prague, Czech Republic; oldrich.n@email.cz (O.N.); vaclav.svorcik@vscht.cz (V.Š.)

**Keywords:** surface modification, laser treatment, plasma exposure, nanoscale design, nanoparticles, tissue engineering, antimicrobial properties

## Abstract

While polymers are widely utilized materials in the biomedical industry, they are rarely used in an unmodified state. Some kind of a surface treatment is often necessary to achieve properties suitable for specific applications. There are multiple methods of surface treatment, each with their own pros and cons, such as plasma and laser treatment, UV lamp modification, etching, grafting, metallization, ion sputtering and others. An appropriate treatment can change the physico-chemical properties of the surface of a polymer in a way that makes it attractive for a variety of biological compounds, or, on the contrary, makes the polymer exhibit antibacterial or cytotoxic properties, thus making the polymer usable in a variety of biomedical applications. This review examines four popular methods of polymer surface modification: laser treatment, ion implantation, plasma treatment and nanoparticle grafting. Surface treatment-induced changes of the physico-chemical properties, morphology, chemical composition and biocompatibility of a variety of polymer substrates are studied. Relevant biological methods are used to determine the influence of various surface treatments and grafting processes on the biocompatibility of the new surfaces—mammalian cell adhesion and proliferation is studied as well as other potential applications of the surface-treated polymer substrates in the biomedical industry.

## 1. Introduction

Biomaterials can be defined as materials that come in contact with biological environments, be it in vitro or in vivo, regardless of their origin. Biomaterials can be metals, ceramics, polymers; they can be fully or partially synthetic, or even completely biological. The way they interact with a biological environment is complex and depends on the physico-chemical properties of their surface [1]. Therefore, to give a biomaterial the appropriate properties for a specific application, it is necessary to modify the part that comes into contact with the environment—its surface. Surface properties such as roughness, morphology, charge, chemical composition, surface energy and wettability all influence the interactions of the biomaterial with biological compounds [2]. These surface properties can be modified by a variety of methods, which include, but are not restricted to, plasma [3] or laser treatment [4], ion implantation [5] and nanoparticle grafting [6]. Each method offers a unique way of surface modulation with its own pros and cons, which are reviewed in detail in this manuscript. 

Biocompatibility of a material is defined as its ability to induce an appropriate answer in a specific situation [7]. Since this “appropriate answer” is defined by the situation in which the biocompatible material is used, it can range from cell adhesion and proliferation on materials used for implanting to complete prohibition of cell growth and coagulation on materials that are used in the bloodstream. The first interaction between a biological system and a biocompatible material is the adsorption of various proteins from the system’s biological fluids onto the surface of the material [8]. The adsorption of proteins to the surface of a polymer depends on its chemical composition as well as its morphology and surface tension [9]. A strongly hydrophilic surface, for example, does not adsorb proteins from blood while a strongly hydrophobic one preferentially binds albumin, due to its high concentration in blood and a favorable diffusion coefficient. In general, the more hydrophilic a surface is and the fewer polar groups it has, the lower the adsorption of proteins it is going to exhibit. For the purpose of cell adhesion, the surface tension needs to be changed by the introduction of new functional groups leading to an appropriate hydrophilicity [10]. It is necessary that, among these newly introduced functional groups, the amino and carboxyl groups are represented, as they are used to bind proteins to the surface of the polymer. Grafting the surface of the polymer with growth factors also facilitates cell adhesion and proliferation [11].

As mentioned above, biomaterials are not limited to a specific group of materials and the choice of the original substrate to be used for subsequent surface treatment depends entirely on the expected application of the final product. Each type of a base material offers different advantages and disadvantages. Metals, for example, exhibit excellent mechanical properties and are thus frequently used as bone or joint replacements [12]. Due to their large expansion coefficient, however, it is necessary to replace them with ceramics or some other material in applications where the increase of volume with rising temperature would prove problematic [13]. Applications where an eventual degradation of the implanted material is desirable, for example in stents or stitches, might call for the use of biodegradable materials, such as certain biodegradable polymers from natural sources [14]. There is a multitude of biomedical applications for the various biomaterials and one of the most widely utilized material groups are polymer substrates.

Polymers come from either natural or synthetic sources and can be relatively easily shaped into structures that range from simple foils to various complex shapes. They offer a wide spectrum of physical-chemical properties due to the large variety in their chemical composition, which makes them interesting candidates for biomedical applications. Their usage is limited by their mechanical properties like tensile strength and Young modulus, which are subpar in comparison with other materials popular in medicine, such as metals or ceramic composites. On the other hand, polymers are very flexible and capable of withstanding large deformations due to the large degree of freedom in the movement of the individual polymer chains. Polymers are semi crystalline materials, and the degree of crystallinity influences their stress-strain behavior, which can range from brittle to highly elastic [15].

Due to their versatility, polymers are widely utilized materials in both scientific and industrial applications. Various polymer substrates offer a broad spectrum of physico-chemical properties, which can be further altered by a variety of surface treatment methods to achieve properties appropriate for each individual application. Surface treatment methods can range from large-scale ones such as chemical modification or UV-lamp treatment, changing the properties of several square meters of surface at a time [16], to precise ones affecting mere square centimeters and changing just a few atomic layers of the substrate, such as laser treatment [17]. Any kind of surface modification will lead to a change of surface chemistry and morphology, which will in turn, more or less noticeably, affect the optical, mechanical, tribological, adhesive, electrical and various other properties of the modified substrate. Such changes should occur only in a small depth away from the surface, while the bulk of the modified polymer and therefore also its properties should remain unaltered [18].

While polymers are promising materials for a multitude of biomedical applications, they are seldom used in their pristine form. Polymers in their pristine state are usually biologically inert and thus some kind of a surface treatment is required to turn them into more advanced materials that induce a specific response in various biological molecules they come in contact with. Modulation of the surface properties of the polymer substrates, such as morphology and roughness as well as physico-chemical composition, is necessary to achieve the desired interaction between the polymer and the biological agent. Appropriate surface modification can alter the morphology of the polymer surface, giving rise to various nanoscale structures which serve as anchor points for specific proteins of the cell membrane, thus improving the adhesion of the cell to the treated surface [19].

The adhesion of cells to a substrate is a two-step process [20]. At first, the cell adheres to the surface thanks to non-covalent interactions (van der Waals, hydrogen bond, electrostatic, polar and ion interactions) between specific molecules in the cell membrane and polar groups on the surface of the substrate. The second step is an adhesion by molecules, such as fibronectin, vitronectin, collagen and laminin, from an extracellular matrix controlled by receptors. The cells attach themselves via integrin receptors located in the cytoplasmic membrane to certain sequences of amino acids [21,22]. The most common sequence of amino acids to which the cell attaches itself is the triplet Arg-Gly-Asp [21]. Due to the complex mechanism of cell adhesion and proliferation, the interaction of the cell with the polymer is dependent on all the physico-chemical and morphological properties of the polymer surface as mentioned earlier, which gives us the option to modulate the surface appropriately for cytocompatibility improvement.

## 2. Laser Treatment

Laser irradiation of solid substrates can, under appropriate conditions, lead to the formation of so-called laser-induced periodic surface structures (LIPSS) [23]. These periodic surface structures come in many shapes and forms, and can be divided into two subgroups based on the relation of their period to the wavelength of the laser radiation. Those that have a period comparable with the wavelength of the incident laser beam are referred to as low spatial frequency LIPSS, while those whose period is much smaller than the wavelength of the laser beam are called high spatial frequency LIPSS. The former are the most commonly observed on polymer substrates while the latter are more often formed on laser-treated semiconductors and metals [24]. 

Low-intensity LIPSS on polymer foils are usually oriented along the main axis of the polarization of the laser beam [25]. For their formation, a good absorption in the wavelength region of the laser radiation by the polymer substrate is necessary. Due to this, LIPSS have mostly been observed on polymers with strongly absorbing groups, such as aromatic rings and systems of conjugated bonds. The exact mechanism of the formation of the ripples is still under dispute, however a broadly accepted theory is that the key to the ripple-forming process is an interference between a primary (incident) beam and a secondary (perpendicularly reflected) beam, which causes local accumulation of energy, resulting in the non-crystalline phase of the polymer substrate being temporarily heated above glass transition temperature and the crystalline one being melted, which allows the material to flow from the high-temperature areas to the lower-temperature ones, thus creating a periodic pattern on the polymer surface [24,26]. The fluence range in which the periodic pattern can be formed differs from polymer to polymer and is furthermore affected by the conditions of the laser treatment. In general, it seems that the bigger an absorption the polymer exhibits in the desired wavelength range, the wider the range of the LIPSS-inducing fluence. For example, using excimer UV laser treatment, LIPSS are formed in a relatively narrow range of roughly 8–11 mJ/cm^2^ on polystyrene (PS) [27], while on polyethylene naphthalate (PEN), they are formed in a much wider range of 6–12 mJ/cm^2^ [28]. Since PS has single benzene ring in its monomer unit while PEN has two, we can assume the higher absorption of UV radiation of PEN, compared to PS, is responsible for the increased fluence range for the LIPSS formation. 

There are several types of LIPSS that can form on polymers and by far the most common ones are ripples. On some polymers, other structures have been observed as well. On PS for example, under certain treatment conditions, the formation of ripples is preceded by the formation of globular structures, which are formed at values of laser fluence insufficient for the formation of fully developed ripples, into which they eventually fuse once the fluence is increased into the ripple-inducing range [29]. The dimensions of these ripples are generally dependent on both the characteristics of the polymer substrate and the conditions of the laser treatment. While the height of the ripples remains relatively constant throughout the entire fluence range in which a homogenous ripple pattern develops on a given polymer regardless of the treatment conditions, the period (width) of the ripples increases with an increasing angle of incidence of the laser beam, according to Equation (1)

(1) $$\Lambda =\frac{\lambda }{n-\sin\varphi }$$

where Λ is the ripple period in nanometers, *n* is the modified refractive index and *λ* is the wavelength and *α* the angle of incidence of the laser radiation [24]. While this equation with a constant *n* is in good agreement with measurements of ripple dimensions obtained on certain polymers such as polyethylene terephthalate (PET) [30], on other polymers, such as PEN and PS, the modified refractive index itself must also be a function of the angle of incidence of the laser beam for the equation to hold true. The dependence of the modified refractive index on the angle of incidence comes from the fact that the index is a material property of the polymer, and thus changes in the structure of the polymer caused by the laser treatment necessarily need to alter the modified refractive index as well. 

**Figure 1:**
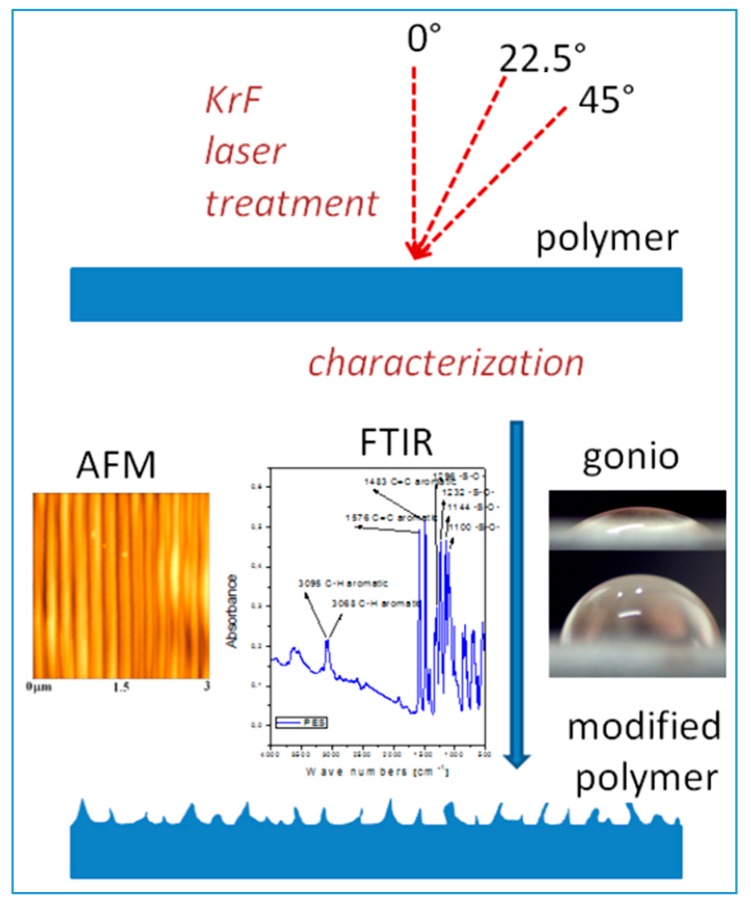
Scheme of the laser treatment process and surface evaluation on polymers such are polyethersulfone (PES), polyetherimide (PEI) and polyetheretherketone (PEEK). The samples treated by KrF laser beam under different angles (perpendicular which corresponds to 0° and sample rotation under the angles of 22.5 or 45°) create various surface morphologies. The modified substrates were characterized by goniometry, UV-Vis and FTIR spectrometry and by AFM [31].

Laser treatment of polymer substrates by fluence well below the ablation threshold increases cell adhesion to the modified surface due to changes in the surface chemistry. Chemical bonds broken by laser radiation lead to a presence of highly reactive radicals on the surface, which quickly react with the surrounding atmosphere. This leads to an oxygenation of the surface and a formation of new functional groups which were not previously present on the pristine polymer, such as oxygen-containing groups like carboxyl and hydroxyl and, to a lesser extent, nitrogen-containing groups like the amino group (Figure 1) [31]. Surface energy of the polymer substrate is also changed by the laser treatment. Such changes in the properties caused by the laser treatment result in an increased adhesion and proliferation of mammalian cells [32]. On PS, for example, the density of the cells on the modified surfaces increases with the duration of the laser treatment to a certain point, where a peak of density roughly 1.9 times greater than that observed on a pristine substrate is reached after 60 s at the fluence of 12.5 mJ/cm^2^ [33]. Increasing the treatment duration further leads to a decrease in the number of adhered cells, which indicates that an excessive number of new functional groups introduced to the surface is contra productive for cell adhesion and proliferation. Increasing the fluence above ablation threshold leads to a loss of the irradiated material. The ablation process can be used, along with chemical surface modification, to create grooves along which the cells align themselves during adhesion [34,35]. The surface along the grooves becomes functionalized by oxygen groups and its roughness increases [36], which leads to an increased cell adhesion in the modified area.

While the pristine surface nonpolar polymers, such as PS, are strongly hydrophobic with high values of contact angle, the laser treatment causes a shift towards hydrophilicity, lowering the contact angle noticeably, regardless of the treatment conditions, such as the angle of incidence or laser fluence. Attenuated total reflection—Fourier-transform infrared spectroscopy (ATR—FTIR) measurements show that the laser treatment introduces a significant number of carbonyl groups into the surface at the expense of both aromatic and aliphatic C-H and C-C groups. In PS, the oxygen content increased from 1.9 at. % (which suggests that the surface is slightly oxidized even in pristine state, since no oxygen is natively present in the styrene monomer) to 21.8 at. % [37]. On polymers that contain oxygen atoms natively, such as polyethylene naphthalate, the increase was less drastic but still very significant [28].

Even though cell proliferation tests using the human embryonic kidney cells HEK-293 cellular line show that there was no noticeable difference between the pristine and the laser-treated PS 1 day after seeding, after 4 and especially after 8 days it became apparent that the surface modification by laser has a positive effect on cell proliferation, due to the changes in surface chemistry and morphology, as the number of cells on laser-treated PS foils increase two- or three-fold compared to the number on pristine PS, yielding results comparable to a commonly used standard, TCPS [37]. 

**Figure 2:**
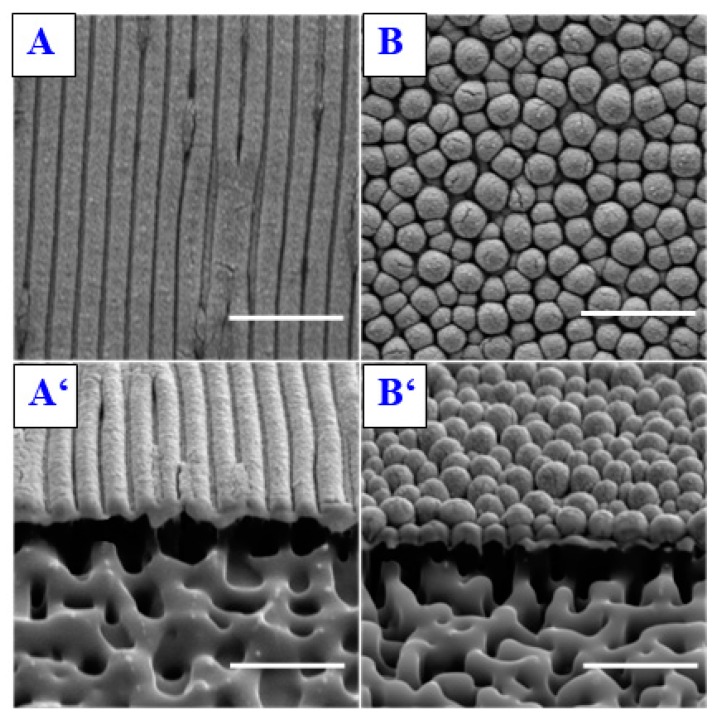
2D SEM images of PES treated by 6000 pulses and laser fluence 8 of mJ/cm^2^ (**A**); 16 mJ/cm^2^ (**B**); bottom images (**A̒**,**B̒**) represent the FIB-SEM cuts of the same samples (**A**,**B**,**A̒**,**B̒**—3 μm × 3 μm) [43].

Another interesting application of laser treatment of polymer substrates for biomedical applications, which was briefly mentioned earlier, is the fact that cells exhibit a preferential orientation along inhomogeneities of the surface morphology [38,39]. For instance, while the human embryonic kidney cells HEK-293 proliferated on pristine PS are spread randomly across the smooth surface, cells proliferated on some of the laser-treated samples of the same polymer exhibit a systematic orientation parallel to the orientation of the ripples. The deciding factor whether the cells do or do not align along the ripples appears to be the ripple period, the width [40]. Cells adhered to samples irradiated perpendicularly (ripple width of 200 nm) exhibit random orientation akin to those adhered to a pristine sample while those adhered to samples irradiated under the 15° angle (270 nm) exhibit preferential orientation in about 50% of cases and the cells proliferated on samples irradiated under the angles of 30° and 45° (340 nm and 430 nm, respectively) are fully aligned along the main axis of the ripples [37]. The degree of the preferential orientation of the cells along the surface structures is affected not only by the dimension of said structures, but also by the type of the seeded cells [41,42]. The Chinese hamster ovary cell line, CHO-K1, for example, while showing results similar to the HEK-293 cell line, seems to prefer structures of larger dimensions. Human myoblasts also show an alignment similar to the CHO-K1 cell line, and the effect of preference for larger surface structures is even more pronounced in rat skeletal myoblasts, which show a preferential alignment only on samples with the with ripples of large periods (430 nm) [37]. Under specific treatment conditions, different types of surface formations, such as globular nanostructures, were observed, e.g., on PES (Figure 2) [43], which may also be applied for cell guidance.

## 3. Ion Implantation

Another way of altering the biocompatibility of a polymer surface is ion implantation, which can increase both the adhesion and the subsequent proliferation of cells [44]. high-energy ions (e.g., in the range 10^13^ to 10^15^ cm^−2^) are separated by magnetic field and accelerated in an electric field before being implanted into the surface of the substrate and their energy is quickly dissipated and causes changes in the structure of the polymer by breaking down macromolecular chains. Breaking of the polymer chains leads to an increased concentration of highly reactive radicals in the surface layer. This leads not only to an increased reactivity of the surface but also to crosslinking of the broken macromolecular chains, formation of double bonds and a release of gaseous degradation products [45,46]. The structures formed in the polymer by ion implantation are similar to those formed by pyrolysis, but they are found only in the surface layers of the polymer instead of in the entire bulk of the material [47]. For biomedical applications, the ions used are either those of noble gasses, which are assumed to be biologically inert, or ions of elements such as oxygen or nitrogen, which can be commonly found in biomolecules [48,49]. For specific applications, ions of cytotoxic elements such as silver can be used to create surface where no cell adhesion takes place [50]. The process of ion implantation is affected by several variables. The depth of penetration is affected by both the weight and energy of the ions. For biomedical applications, low energies in the range of tens of keV are often utilized since it is assumed that cells only interact with the uppermost layers of the material and thus shallow penetration depths are sufficient [51]. 

For the adhesion and proliferation of mammalian cells, good anchoring to the substrate is necessary. For that, appropriate physico-chemical properties and morphology of the surface are needed. Pristine polymers are usually hydrophobic or negatively charged, which makes cell adhesion difficult. Ion implantation allows the modulation of surface properties of a polymer, including its biocompatibility. The increase in biocompatibility can be attributed, among other things, to an increased polarity of the modified surface and the presence of oxygen-rich groups, as well as to an increase in its roughness, which leads to an increase in surface area [52]. The adhesion of cells to a substrate is mediated by bonding via electrostatic forces but also via specific molecules of the extracellular matrix [53]. 

Ion diffusion into the surface layers of the modified polymer increases conductivity and biocompatibility. Lower ion doses lead to the formation of oxygen-containing groups and an increase in free volume, thus facilitating further penetration into the modified layer, creating a slightly porous structure. On the contrary, higher doses lead to more prominent crosslinking and the formation of two- and three-dimensional structures, accompanied by a decrease of free volume, which are harder to penetrate by the subsequent ions [54]. It is, therefore, possible to modulate the depth of penetration not only by the energy of the implanted ions but also by their density. Another important aspect is the size of the individual atoms. For example, small atoms such as fluor aren’t incorporated into the modified layer and are released with other gaseous byproducts, while larger atoms such as iodine are incorporated well and remain in the polymer substrate [55]. The implanted atoms are bound either to the highly reactive free radical sites or to the newly formed double bonds [56]. Given equal energy, lighter ions have a higher mean distance range of penetration into the polymer in comparison to heavier ones, which are implanted closer to the surface of the modified substrate. Since ions always penetrate deeper than the uppermost surface layer regardless of weight and energy, it is a safe assumption the adhesion of cells is dependent not only on the very surface of the polymer but also on the physico-chemical properties of a certain depth range of the surface layers [57]. 

Ion implantation also significantly changes the morphology of the treated surface. Argon and oxygen ions, for example, increase the roughness of a surface proportionally to the dose, and lead to the formation of a fibrous structure. However, in some cases, the earlier mentioned crosslinking and the decrease of free volume caused by higher doses of ions can lead to a decrease in surface roughness in comparison to the effect of lower ion doses [58]. The morphology of a surface is crucial when it comes to cell adhesion. Cells have been known to align themselves along grooves formed during surface treatment [59]. Increase of surface roughness on polymers that are very smooth in their pristine state often leads to an improved cell adhesion. In general the effect of surface roughness and morphology is dependent on both the polymer substrate and the type of seeded cells [60]. 

The electric conductivity increase caused by ion implantation is attributed not only to the bond breaking of the macromolecule chains by the implanted ions and the subsequent formation of free radicals and conjugated double bonds, but also to the presence of conductive dopants in the treated surface layers. Carbonization and graphitization, which also occurs during ion implantation given sufficient energy and ion doses, also positively impacts the electric conductivity of the surface [61]. Interactions between the surface charge and the charge on the seeded cells are an important factor for proper adhesion. Specific sites with a negative charge on the cellular membrane are utilized in both recognition and adhesion processes when a cell gets into a contact with a foreign surface. Whether a slightly negative or a slightly positive charge will facilitate adhesion is dependent on the type of the seeded cells. Furthermore, slightly charged groups influence the sorption of specific proteins of the cellular membrane responsible for proper adhesion [62].

The changes of the wettability of the polymer substrate caused by ion implantation, affected by the modulation of chemical composition, roughness and morphology of the surface layers also have a noticeable effect on its biocompatibility [63]. While ion implantation generally increases the polarity of the surface of nonpolar polymers due to the formation of oxygen-rich groups on the active sites created by breaking the macromolecular chain, the implantation of polymers with polar surfaces has the opposite effect. This is attributed to the degradation of polar groups, which were already present in such substrates [19]. Since an appropriate ion dose decreases the mobility of the macromolecular chains due to increased crosslinking, the reorientation of the polar groups into the bulk of the material observed on plasma-treated samples, which causes an increase in hydrophobicity as polymer substrates age, is suppressed [64]. This is useful for the creation of lasting polarity changes in the surface of highly hydrophobic polymers necessary for appropriate interaction of such nonpolar substrates with various types of mammalian cells. While the polarity of the polymer substrate is an important factor in its interaction with seeded cells, the specific values of wettability of the surface are dependent on the type of the seeded cells.

When it comes to biocompatibility increase caused by the changes of the surface chemistry of the treated samples, it is the biogenic elements such as C, N and O and their functional groups, which are attributed for it [65]. The presence of amino acids, for example, has been observed to be directly proportional to the adhesion and spreading of seeded cells. The positively charged amino groups take part in the interactions with the negatively charged bonding sites of the cell membrane. The oxygen-rich groups also facilitate the adhesion of cells to the modified surface [66]. Certain mammalian cells can also respond positively to a surface where both hydrophobic and hydrophilic microscopic domains are present, since domains with different wettability and chemical structure are preferred by different proteins. Such domain structure can be achieved by selective ion implantation through a contact mask and leads to an organized mosaic of adsorbed proteins [67]. 

## 4. Plasma Treatment

During plasma treatment, a noticeable loss of material may occur. The thickness of the exposed or treated layer is dependent both on the polymer substrate and on the power and duration of plasma treatment. Chemical and physical structure of the polymer plays a key role when it comes to etching. Polymers with a higher degree of crystallinity exhibit a lower etching rate than the more amorphous ones, due to the greater structural integrity of the crystalline phase. Molecular weight and the orientation of the polymer chains also play a role in the rate of etching [68]. The etching rate and the changes in morphology caused by plasma treatment are naturally dependent not only on the duration of the treatment, but also on the power used [69].

Water contact angle measurements taken right after a plasma treatment generally show a significant decrease of the water contact angle regardless of the treatment duration, depending on the type of the polymer [70]. The surface which was originally strongly hydrophobic thus obtains hydrophilic properties, which are, however, only partially retained as the sample ages. The slow reversal of the effects of the plasma treatment on the polarity of the surface is caused by the reorientation of the oxygen-rich polar groups, formed on the ablated surface during the plasma treatment, into the bulk of the material, thus altering the surface chemistry by decreasing the oxygen content [71]. The wettability of the plasma-treated polymer substrates depends on the plasma power, bias voltage, type of gas and gas flow. The amount of oxygen adsorbed by the modified polymer surface during and after the plasma treatment is also dependent on the motility of the macromolecular chains, which also influences both the crosslinking on the surface and the crystallinity of the whole polymer [72]. For example, UHMWPE, a polymer with highly motile macromolecular chains [73], exhibits a greater increase in atomic oxygen content after the plasma treatment than PEN, a polymer whose chains are much more rigid due to the presence of the naphthalene group [74]. The topic of wetting has received tremendous interest from both fundamental and applied points of view. Wettability studies usually involve the measurement of contact angles as the primary data, which indicates the degree of wetting when a solid and liquid interact. Consider a liquid drop resting on a flat, horizontal solid surface, the contact angle is defined as the angle formed by the intersection of the liquid-solid interface and the liquid-vapor interface (geometrically acquired by applying a tangent line from the contact point along the liquid-vapor interface in the droplet profile). Small contact angles (<90°) correspond to high wettability, while large contact angles (>90°) correspond to low wettability. When contact angle is small the liquid spreads on the surface, while a large contact angle is observed when the liquid is not spread.

Plasma treatment also leads to changes in surface roughness of the modified polymers. While the roughness increase of some polymers might be barely noticeable (LDPE), on others, the increase is much more significant (HDPE). On other polymers still, the plasma treatment can actually lead to a decrease in surface roughness (UHMWPE) [73]. The various degrees of changes to the surface roughness caused by plasma modification are interlinked with the above mentioned ablation rate. Since the amorphous phase is ablated at a greater rate than the crystalline one, plasma treatment gives rise to various crystalline structures after the ablation of the amorphous phase occurs on the surface of polymer substrates where both phases are somewhat equally present, which results in an increased surface roughness. On the other hand, on polymers where one phase is predominantly present, the material is ablated evenly which can lower the roughness of the surface [75]. 

**Figure 3:**
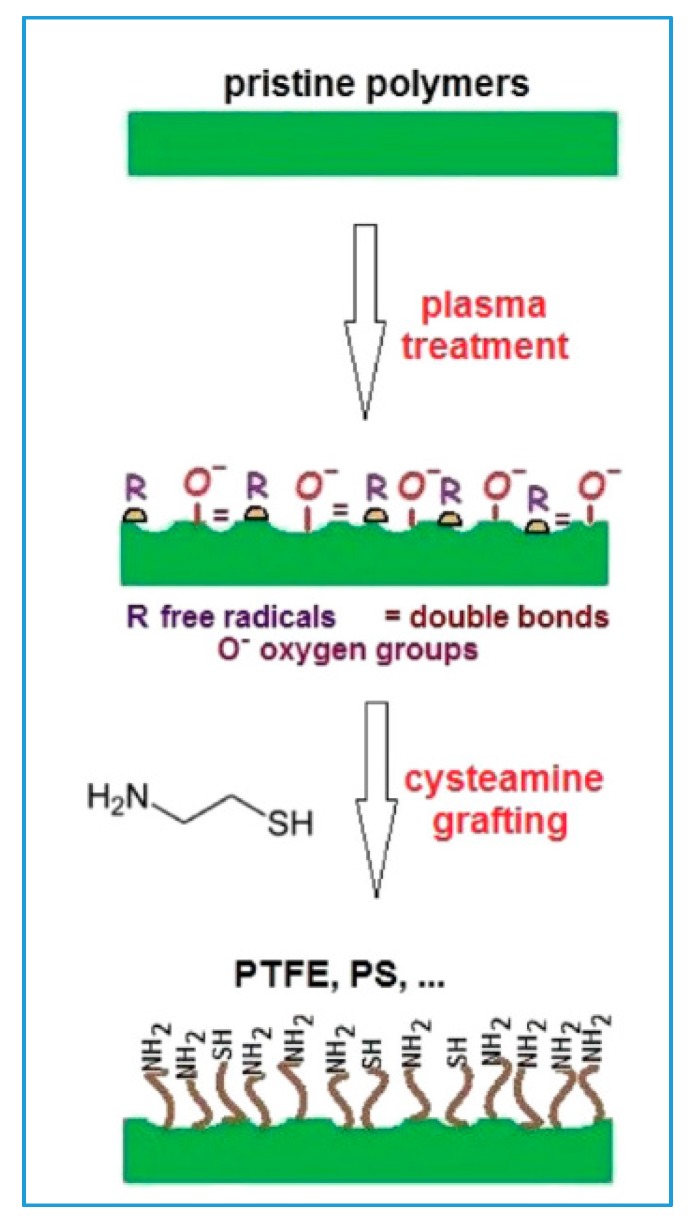
Scheme of grafting of cysteamine on plasma treated PTFE and PS [79].

Plasma treatment also leads to a drastic change of the zeta potential, which depends on the chemistry, polarity and charge of the surface, as well as its morphology and thus its roughness. The polar groups introduced to the surface by the plasma treatment increase the zeta potential of the modified samples [76]. Depending on the chemical composition of the polymer in its pristine state, the values of the unaltered surface range from strong hydrophobicity in the case of nonpolar polymers to hydrophilicity of polar ones. The increase in zeta potential is dependent on the duration of the treatment—the longer the samples are treated, the greater the increase [77]. The influence of the power used on the change of the zeta potential is much less significant. Interestingly, enough, contrary to the goniometric measurements, aging has next to no influence on the zeta potential as the charge remains stable even a month after the modification for some specific cases [78]. Since major proteins and cellular membranes have a slightly negative charge in an environment with physiological pH, the plasma-induced shift towards a positive charge on the polymer surface facilitates the adhesion of mammalian cells, which may be also affected by the grafted groups on the polymer surface (Figure 3) [79]. 

**Figure 4:**
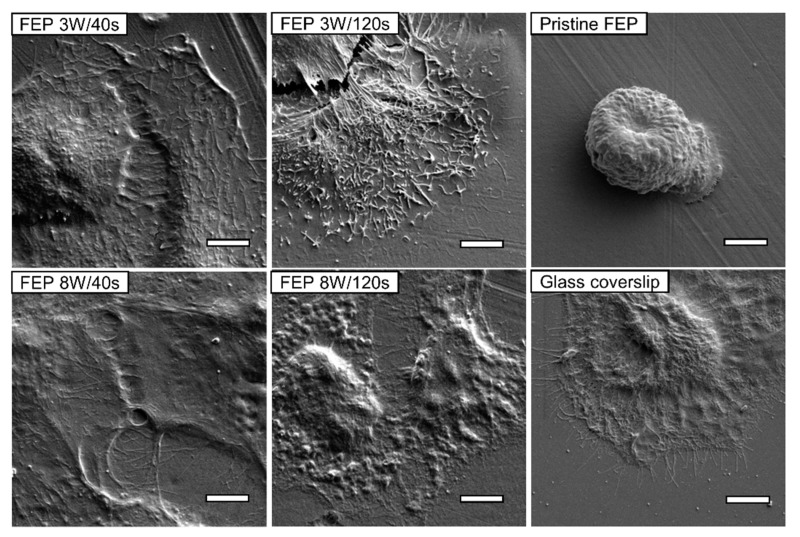
Scanning electron microscopy images of human keratinocytes (HaCaT) 24 h after seeding on plasma treated FEP, pristine FEP and glass coverslip. The power of plasma (3 and 8 W) and treatment durations (40 and 120 s) are given for each image. Scale bar is equal to 5 µm [80].

Testing the biocompatibility of the various plasma-treated polymer substrates to different cell lines demonstrates the necessity of modifying each substrate in a specific way for obtaining the appropriate biological response for the desired application. In the case of PE, tests of mammalian cell adhesion using rat vascular smooth muscle cells (VSMC) and mouse fibroblasts (L929) showed that the plasma treatment leads to a significant increase of the biocompatibility of the surface for all three polymer modifications (LDPE, HDPE and UHMWPE). The parameters of the plasma treatment for optimal biocompatibility for these cell lines are dependent on the substrate. While the shortest treatment time of 120 s leads to the highest increase in the number of adhered cells for both LDPE and HDPE, on UHMWPE the longer treatment time of 240 s yields the best results. When compared with TCPS standard, the plasma-treated PE substrates show a significantly higher number of adhered L929 cells while the VSMC cell adhere in much greater numbers to the standard tissue polystyrene. Furthermore, metabolic assay tests show that the viability of the L929 cell line is much lower on the PE-based substrates compared to the standard TCPS. This stresses that the number of adhered cells alone is not a sufficient to evaluate the cytocompatibility of a given material [73]. In comparison with the behavior of the VSMC cellular line on the PE-based substrates, the results of PEN are more promising. All the modified samples exhibit a much higher number of adhered cells 7 days after seeding in comparison to TCPS. The viability of the proliferated cells is very high on all plasma treated samples, exhibiting values comparable with the viability measured on the TCPS standard. Even the cells proliferated on pristine PEN exhibit similar viability despite their lower numbers, which suggests that PEN is a material with a good potential for biomedical applications, and even a quick plasma treatment at low power greatly increases the attractiveness of its surface to mammalian cells [74]. Argon plasma treatment with low power (up to 8 W) significantly influences the cell morphology, which can be demonstrated e.g., on plasma treated FEP in comparison to a pristine foil (Figure 4) [80].

**Figure 5:**
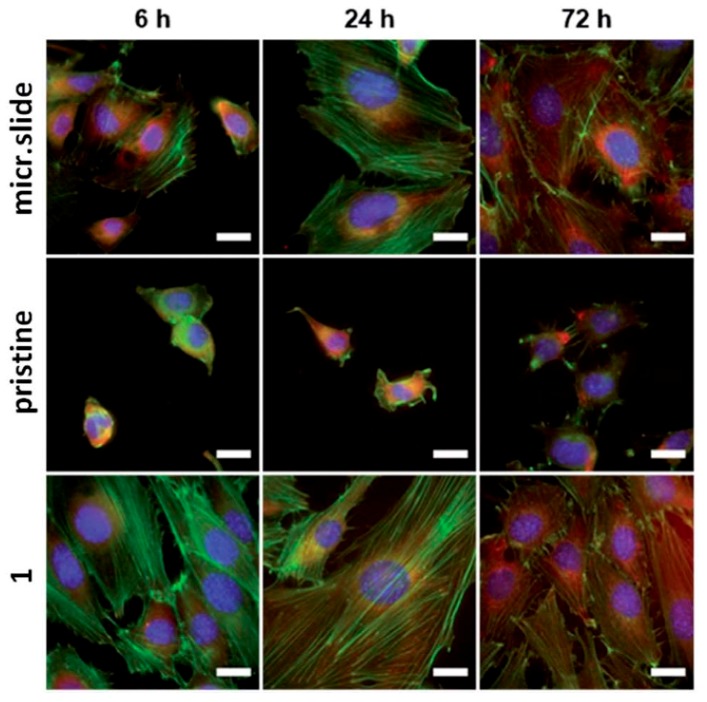
Immunofluorescence microscopy images of NIH 3T3 cells growing on a pristine polymer, control and plasma treated samples—1 (100 W, 240 s, O2/Ar), for 6, 24, and 72 h. F-actin of cell cytoskeleton in green (phalloidin-Atto 488), nucleus in blue (DAPI), and talin 1 was visualized by antibody labeled with Atto 647. The scale bar is equal to 20 μm.

The high-power plasma was used as a tool for roughening of the surface and surface chemistry changes with aim of biocompatibility improvement. The higher plasma power deepened the profile of the surface, which was associated with increased roughness. It was confirmed that both Ar and O_2_/Ar plasma induced significant changes of surface contact angle after aging procedure of FEP foil. The proliferation of mouse embryonic fibroblast cells (NIH 3T3) cells was several folds higher on plasma treated PMP if we compare the results with pristine PMP. The rate of cell growth on treated PMP matrices resembled to that of TCPS. Also the different type of cell shape on the basis of plasma exposure was determined. The cells on pristine PMP exhibited rounded shape, were not spread and lacked typical actin protrusions (Figure 5) [81]. Viability of the adhered cells is strongly influenced by the hydrophilicity, chemical changes and surface roughness of the substrate. This is especially true in the case of osteoblasts, where a high surface roughness has a positive effect on their metabolic activity [82]. 

Plasma processing may be used to fabricate superhydrophilic or superhydrophobic polymeric surfaces by means of oxygen plasma etching of organic polymers, such as poly(methyl methacrylate) (PMMA) [83] and poly(ether ether ketone) (PEEK) [84]. The plasma modification of polymeric substrates has been used for many potential applications in biology and biochemistry. The modified surfaces may enhance the protein absorption [85], induce superhydrophobic paper surfaces by using atmospheric pressure plasma etching [86], they may be applied for biomolecule immobilization and environmentally stable super hydrophobic and superoleophobic behavior [87,88] or lab-on-a-chip applications [89]. Biomedical applications of natural-based polymers combined with bioactive glass nanoparticles have been reported recently [90], the influence of silver doped bioactive glass nanoparticles on antibacterial bioadhesive layer for orthopedic applications was confirmed as well [91]. Immobilization of biomolecules on surfaces may be considered as the first step for the construction of a large variety of functional substrates, which include microarrays, cell arrays, microfluidics for diagnostic purposes, biocompatible surfaces for medical applications such as implants, and many more [92]. Plasma treatment can be applied for initial surface activation, which further enhance the bonding of different molecules or more complex compounds [93]. A biomolecule is generally accepted to be “immobilized” to a solid support if it is either physically adsorbed to the support via van der Waals forces, hydrogen bonding or electrostatic interactions or covalently attached to active surface groups and cannot be removed from the support by simple methods such as washing with buffers. Energetic ion bombardment during plasma modification and polymerization processes were shown to be effective for promoting the robust surface attachment of protein molecules with extended longevity of activity compared to protein layers on untreated and plasma treated (without ion bombardment) controls. The long-term retention of bioactivity both in solution and after freeze-dried storage appears to be correlated with the long-term retention of hydrophilic character observed on the ion treated surfaces [94]. Covalent immobilization is considered to support higher activity of biomolecule, reduced nonspecific adsorption, and greater stability of immobilized biomolecules compared to that by physical adsorption [95].

## 5. Nanoparticle Grafting

Nanostructured surface can facilitate cell adhesion by providing focal points for the filopodia to attach themselves to, therefore mimicking the important parts of the morphology of an extracellular matrix [96]. There are a multitude of choices when it comes to the selection of the material for the creation of the nanoparticles, with each offering its own distinct advantages and disadvantages. Among some of the most popular materials are the various noble metals, each of which has unique properties. For example, silver nanoparticles have antimicrobial properties and are thus often utilized on substrates where the interaction with the biological environment is undesirable [97], while gold nanoparticles rarely induce an allergic response and are believed to not be cytotoxic. Gold nanoparticles furthermore exhibit good biocompatible properties and are thus a good candidate for various biomedical applications in immunology, nanomedicine and biotechnology [98,99]. Carbon-based nanoparticles are also an interesting option as there are several types of nanoscale carbon structures with promising biomedical applications due to their unique physico-chemical properties. The most commonly studied ones are carbon nanotubes, diamond-like carbon and carbon nanodiamonds. Carbon nanotubes can be used as systems for drug delivery, or, due to their very high electrical conductivity can improve the healing of damaged nerve fibers. Their good mechanical properties also make them an interesting candidate for the creation of polymer composites utilized as bone replacements. Similarly, the excellent mechanical properties of diamond like carbon make it popular as a coating for various implants [100,101,102,103].

During the process of creation of nanoparticles for the purpose of grafting on the surface of polymers for various biomedical applications, there are multiple variables that need to be controlled, such as shape, size and its distribution. One of the most important and difficult variables to control is the dispersion of the nanoparticles. Nanoparticles have very high surface energy, which makes them inclined to agglomerate, and thus an appropriate medium for the creation of a nanoparticle solution or an additional surface treatment is often required [104]. The carbon nanoparticles, for instance, can be functionalized by various amino groups, which improve their wettability and make the preparation of a water suspension easier due to the suppression of their tendency to agglomerate [105]. 

Grafting of a polymer substrate is often a multi-step process, where the physico-chemical properties and the morphology of the surface must first be modified to ensure appropriate interactions and sufficient bonding sites for the grafted nanoparticles or biomolecules [106]. There are various degrees of surface activation. In the case of PET foils grafted by noble metal nanoparticles (Pt and Pd), for example, the surface was first treated by plasma to create a sufficient number of bonding sites. The active sites created by the plasma treatment were consequently occupied by biphenyl-4,4’-dithiol (BPD), which is used as a binding agent to anchor the Pt and Pd nanoparticles to the polymer substrate. Finally, mammalian cells were grafted to the newly created surface [107].

Bond-breaking of the macromolecular chains caused by the plasma pre-treatment leads to the formation of highly reactive free radicals on the surface of the modified polymer. These radical active sites are quickly attacked by oxygen molecules from the atmosphere, forming various oxygen-containing groups and thus increasing the polarity of the surface and decreasing its water contact angle, i.e., increasing its wettability. The wettability changes are then slowly reverted as the samples age and the polar groups on the surface of the polymer reorient themselves into the bulk of the material [108]. After a sufficiently long aging time, the values of the contact angle might even surpass the original values of the pristine substrate, in the case of aromatic polymers such as PET [109] or PEN [74]. The degree to which the contact angle rises during the aging process is dependent not only on the chemical structure of the polymer substrate, but also on both the power of the plasma discharge and the treatment duration [110]. In the case of PE, which contains no aromatic cores in its structure, the shorter duration treatments lead to a greater overall decrease of the contact angle, with the 50 s treatment exhibiting the lowest contact angle of them all. From that point, increasing the treatment duration leads to an increase in the contact angle, with samples treated for the duration of 200 s and higher eventually exceeding the contact angle of a pristine PE. Once the plasma pre-treated surface is grafted by BPD and/or by Au nanoparticles, the contact angle decreases significantly and its dependence on the duration of the original plasma treatment becomes less steep [111].

**Figure 6:**
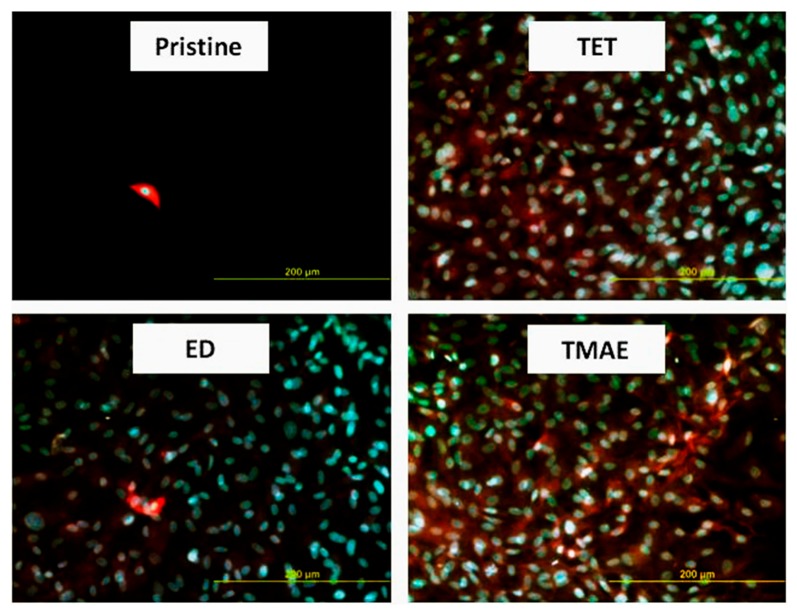
Photographs of VSMCs proliferated (6th day) on pristine HDPE and CNPs (ED, TET, TMAE) grafted HDPE. Chemicals used are ethylenediamine (ED), diethylenediamine (DET), tris[2-(methylamino)ethyl]amine (TMAE).

Material loss caused by the plasma pre-treatment can result in various structures appearing on the ablated surface due to the crystalline and amorphous phases of the polymer being ablated at different rates [112]. In the case of PE [111], for instance, the pre-treatment gives rise to lamellar structures on the ablated surface, increasing its roughness while on PET [113], globular structures have been observed after the plasma modification. The morphology of the plasma-activated polymer substrates is further altered by the subsequent anchoring of bonding agents and the grafting of the nanoparticles. For example, grafting the plasma-treated PE surface with BPD lowers the roughness slightly and makes the lamellar structures narrower while the subsequent Au nanoparticle grafting leads to a formation of gold nanoclusters and an increase in roughness to pre-BPD-bonding levels [111]. Similar effect of roughness increase has also been observed on PET and HDPE grafted by functionalized carbon nanoparticles [113], where the positive effect of carbon nanoparticles on cell proliferation was observed (Figure 6).

**Figure 7:**
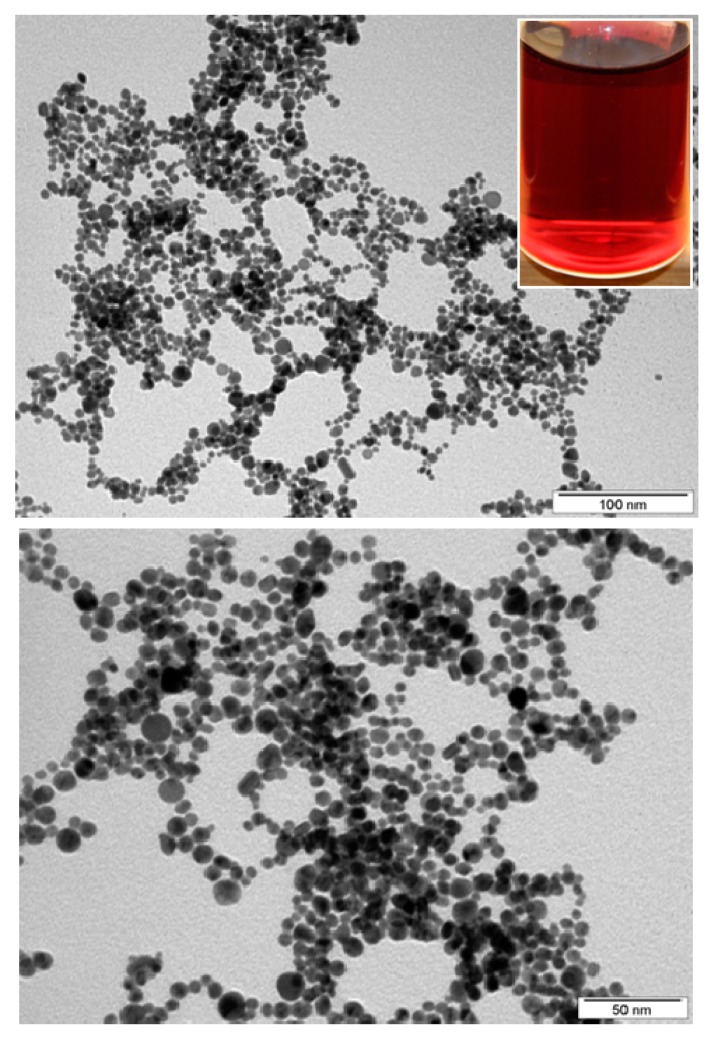
TEM images of Au nanoparticle solutions in PEG/H_2_O obtained by cathode sputtering, prepared at 30 mA and 300 s with ratio 1:9.

The amount of nanoparticles bound to the modified surface depends not only on the plasma and chemical pre-treatment but also on the type and material of the grafted nanoparticles. Nanoparticles have rather different preferences when it comes to the chemistry and morphology of the surface—higher concentration of sulfur on substrates pre-treated by BPD, for example, which suggest more binding spots for the nanoparticles, does not necessarily mean an increase in their surface concentration. On PET, the Pt nanoparticles exhibited a preference towards longer plasma pre-treatment times while for the Pd nanoparticles, the opposite was true [107]. The preferential bonding of the noble metal nanoparticles to the thiol groups of the BPD anchored to the plasma-treated polymer surface was confirmed by FTIR analysis of the absorption bands corresponding to the C-SH bond of a thiol group anchored to the surface and the C-S-Pd and C-S-Pt bonds of the respective nanoparticles bonded to the other, free thiol group [107]. Similar result was achieved when utilizing BPD to anchor nanoparticles to the surface of PE [111]. The plasma pre-treatment, as well as the final grafting of the noble metal nanoparticles also leads to a significant change in zeta potential, due to the change in the surface charge of the polymer cause by the increase in polar groups in the surface layer [114]. Pt and Pd nanoparticles caused a significant shift towards a positive charge [107], while Au nanoparticles (Figure 7), on the other hand, cause a dramatic decrease well below the values of the pristine polymer substrate, greatly increasing the conductivity of the grafted surface, which is also known to improve biocompatibility [111]. 

**Figure 8:**
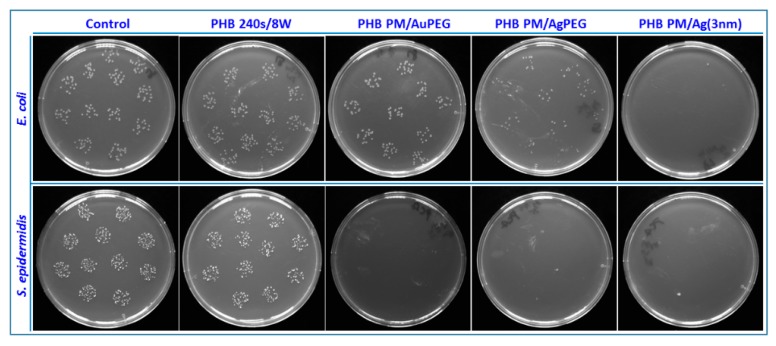
The photographs of *E. coli* and *S. epidermidis* colony forming units on the samples: control sample, plasma modified sample with the power of 8 W and time 240 s (PHB 240 s/8 W), and then plasma modified sample consequently: (i) immersed to Au/PEG/H_2_O solution (PHB PM/AuPEG); (ii) immersed to Ag/PEG/H_2_O solution (PHB PM/AgPEG) or sputtered with silver of the effective thickness of 3 nm determined by gravimetry (PHB PM/Ag, 3 nm) [121].

As mentioned earlier, various nanoparticles are suitable for different biomedical applications [115,116,117]. Furthermore, the change of surface properties such as wettability, roughness, charge, conductivity and others, caused by the plasma pre-treatment and the subsequent nanoparticle grafting, which would individually refer to a biocompatibility improvement might not actually have an overall positive effect in the end. The interaction of the nanoparticles themselves with the biological environment also affects the adhesion and proliferation of cells in a significant way and different cells exhibit varying degrees of affinity towards different nanoparticles [118]. For instance, cytocompatibility tests done on the nanoparticle-grafted surface of PET using the fibroblast mouse cells (L929) and human bone osteosarcoma (U-2 OS) cellular lines using TCPS as a standard show that PET functionalization by Pd nanoparticles actually leads to a decrease far below the number of cells observed on pristine PET due to the cytotoxicity of Pd, while grafting with Pt ones increases the number of cells, exceeding even the number proliferated on the TCPS. The U-2 OS cellular line, however, exhibits a rather different behavior on the same substrate. For this cell line, both Pt and Pd are cytotoxic [119]. On the contrary, biocompatibility tests using the VSMC line on Au-grafted PE show that the PE functionalized by BPD-anchored Au nanoparticles has excellent properties for cell adhesion and proliferation [111]. VSMC also exhibit good affinity to surface grafted with carbon nanoparticles, especially on PET. On carbon nanoparticle grafted HDPE, the amount of proliferated cells is slightly lower than on a sample treated solely by plasma, but still much higher than the amount observed on a pristine substrate [113]. On the other hand, silver nanoparticles greatly enhance both the antibacterial and the cytotoxic properties of a polymer substrate when grafted to its surface (Figure 8), thus allowing for applications where the suppression of cell adhesion to a biomaterial is necessary [120].

Even though the techniques of plasma, laser or ion beam modification have been well known for several years, still a lot of perspectives can be found in this very interesting and wide area of research. We think that there are still some significant challenges, mostly based on the development of new basic materials (polymers, metals and others), which can be further processed and new techniques of the surface treatment based on different plasma, laser or ion beam sources in combination with different grafting procedures. We think that also 2D and 3D printing of e.g., polymer materials with unique properties, which would be able to specifically react with e.g., wavelength of laser beam or which would have incorporated specific chemical groups in the polymer chain have a high potential. The synergy on newly developed materials in combination with enhanced techniques of surface modification and grafting procedures can bring new materials with high potential as tissue replacements, organ replacements, biosensors, antibacterial materials or materials for specific applications.

## 6. Perspectives and Challenges 

Polymers can be described as important cell carriers for tissue engineering. In their pristine state, however, they do not often possess the properties that are appropriate for their application as biocompatible materials. We have shown that polymer surface modifications, laser and plasma treatment, ion implantation and nanoparticle grafting play important role in tissue engineering applications. Surface treatment is able to induce changes of the physico-chemical properties, morphology, chemical composition and biocompatibility of a variety of polymer substrates. By the application of laser beam, periodic nanopatterns of various types can be constructed and thus both the growth and orientation of the adhered cells can be controlled. Introducing new elements to the surface layers of a polymer substrate by ion implantation has also proved to have a positive effect on its biocompatibility. We think that there are still some significant challenges, mostly based on the development of new basic materials (polymers, metals and others), which can be further processed and new techniques of the surface treatment based on different plasma, laser or ion beam sources in combination with different grafting procedures. We think that also 2D and 3D printing of e.g., polymer materials with unique properties, which would be able to specifically react with e.g., wavelength of laser beam or which would have incorporated specific chemical groups in the polymer chain have a high potential. The synergy on newly developed materials in combination with enhanced techniques of surface modification and grafting procedures can bring new materials with high potential as tissue replacements, organ replacements, biosensors, antibacterial materials or materials for specific applications.
